# Image-guided surgery and therapy

**DOI:** 10.2349/biij.3.1.e7

**Published:** 2007-01-01

**Authors:** TZ Aziz

**Affiliations:** Oxford Functional Neurosurgery, Nuffield Department of Surgery, University of Oxford, United Kingdom

I am pleased to see the publication of this very novel issue. It brings together in one volume the role modern imaging has to play in surgery and radiotherapy. Certainly in functional neurosurgery imaging has transformed the way it is performed.

In its first blossoming in the 1950s, patients were fixed in a stereotactic frame, contrast was injected into the ventricles and x-rays were taken of the patient’s head in the frame from AP and lateral projections. One then had to make corrections for the degree of magnification and divergence of the x-ray beam. Following this, the anterior and posterior commissure were identified on the ventriculogram and the mid-commissural point and its co-ordinates calculated. Having done so, the surgeon referred to an atlas based upon cadaveric studies to calculate the position in the frame with the highest probability of hitting the target. In those days, the motor thalamus or pallidum was identified for Parkinson’s disease and tremor [1]. Morbidity after contrast ventriculography was not unusual [2].

Although surgery was effective in abolishing tremor, there were many side effects as the lesions were imprecise by nature. Alleviation of pain with deep brain surgery had even more side effects as the targets, the peri-acqueductal gray area and sensory thalamus, lay in pathways that were also involved in emotion [3].

Deep brain stimulation though studied in the 1950s was never used long term because of technical limitations. Also, with plain x-rays and ventriculography it was never precise where the electrodes were placed [4]. Ventriculography was also poorly tolerated because it was associated with headaches and nausea.

The introduction of computed tomography (CT) scanning came about at a time when functional neurosurgery had all but stopped. However, for the first time surgeons were able to visualise tumours and the frame emerged again as a useful tool for biopsying tumours, which was in fact the first form of image-guided neurosurgery [5]. It was also used for stereotactic craniotomies. However, CT scans could not visualise deep brain structures apart from the AC and PC. Therefore in performing functional neurosurgery, CT scanning eliminated the need for contrast ventriculography which made such surgery far more tolerable [6]. It also meant that the accuracy of localisation was no better than CT scanning either.

With better understanding of the physiology of movement disorders and pain, new and old targets became far more relevant for disease alleviation. Accurate placement of lesions and electrodes became paramount. Modern magnetic resonance imaging (MRI) scans were able to visualise target areas clearly in the brain so probabilistic atlases were no longer vital and distortions of the MRI space could be corrected by various methodologies. This made localisation more accurate and to a large degree made prolonged sessions of micro-electrode recordings unnecessary [7].

The article by Widman eloquently describes the development of image-guided surgery from a point target frame based method to the volumetric techniques that are in common use today. This development of radiological images used interactively in 3D space has transformed modern neurosurgery and made procedures safer and quicker [8]. The use of such technology to guide tools like the operating microscope and actual surgical tools has largely supplanted stereotactic frame based surgery.

The use of new technologies has also allowed us to gain better insights into how we can modify brain function in pathological states. Today we can implant deep brain electrodes to alleviate movement disorders, pain, epilepsy and psychiatric disorders. The electrodes can be externalised to record deep brain activity and relate it to alteration of physiological function [9]. Careful physiological studies can reveal surprising new indications for the therapy [10] as the paper by Pereira *et al* has shown. In a study of the physiological changes that accompany pain alleviation, it emerged that dorsally-placed electrodes raise blood pressure while ventrally-placed ones decrease blood pressure. Given the vast numbers of patients with poorly controlled blood pressure despite best medical therapy, we may be looking at the emergence of a new indication.

Patients with chronically implanted deep brain electrodes are not yet generally amenable to functional MRI (fMRI) scanning due to safety issues [11] though there are some studies [12]. To date, the only accepted methodologies were single photon emission tomography (SPECT) and positron emission tomography (PET) [13] scans with limitations of time and resolution. However, since the 1990s magnetoencephalogram (MEG) scanning has been extensively used to map out cortical activity by measuring changes in the magnetic fields [14]. Recently, paradigms have been developed to study the activity in deep brain structures and also to render this information in 3D space with better spatial and temporal resolution than PET, SPECT or fMRI. More importantly, this can be done in patients with the stimulator on and off, and can eliminate artefacts of stimulation. The work by Kringelbach *et al* has demonstrated this for neuropathic pain and in this issue Wray and Kringelbach report their study in a case of cluster headache which responded to deep brain stimulation in the postero-infero-medial hypothalamus.

These are landmark developments. Image guidance has made major brain tumour surgery safer. The use of diffusion tensor imaging to map out connectivity of brain pathways and the use of MEG scanning to study brain activation i.e., on and off stimulation, and correlating them all with field potential recordings from the deep brain electrodes and changes in physiological functions i.e., movement disorders, pain psychiatric disorders, etc., will unravel mysteries of brain function in a way never possible before.

## ACKNOWLEDGEMENTS

Oxford Functional Neurosurgery is supported by the MRC, Charles Wolfson Charitable Trust and Norman Collisson Foundation.
